# A Novel Hierarchical LATER Process Model: Evaluating Latent Sources of Variation in Reaction Times of Adult Daily Smokers

**DOI:** 10.3389/fpsyt.2019.00474

**Published:** 2019-07-05

**Authors:** Nicole J. Roberts, Zita Oravecz, Briana N. Sprague, Charles F. Geier

**Affiliations:** ^1^Department of Human Development and Family Studies, The Pennsylvania State University, University Park, PA, United States; ^2^Institute for CyberScience, The Pennsylvania State University, University Park, PA, United States

**Keywords:** smoking, cognition, cognitive model, abstinence, Go/NoGo task

## Abstract

Reaction time data from cognitive tasks continue to be a key way to assess decision-making in various contexts to better understand addiction. The goal of this paper is twofold: to introduce a nuanced modeling approach for reaction time data and to demonstrate the novel insights it can provide into the decision processes of nicotine-dependent individuals in different contexts. We focus on the Linear Approach to Threshold with Ergodic Rate (LATER) model, which is a cognitive process model that describes reaction time data in terms of two distinct aspects of cognitive functioning: speed of information accumulation (“accretion”) and threshold amount of information needed prior to execution (“caution”). We introduce a novel hierarchical extension to the LATER model to simultaneously account for differences across persons and experimental conditions, both in the accretion and caution parameters. This approach allows for the inclusion of person-specific predictor variables to explain between-person variation in terms of accretion and caution together with condition-specific predictors to model experimental condition manipulations. To highlight the usefulness of this model, we analyze reaction time data from a study on adult daily cigarette smokers. Participants performed a monetary incentivized Go/No-Go task during two testing sessions, once while following their typical smoking patterns and again following 12 h of verified smoking abstinence. Our main results suggest that regardless of trial type, smokers in a period of abstinence have faster accretion rates, and lower caution thresholds relative to smoking as usual.

## Introduction

A fundamental goal of psychiatry and neuroscience research is to understand how and why humans make decisions and behave as they do across various contexts. In particular, work aimed at understanding how exposure to addictive substances like nicotine impacts and alters decision-making is of considerable interest. The examination of reaction time data acquired from cognitive tasks continues to be a major way to assess decision-making, yet traditional analysis of such data (e.g., evaluation of group-level means and variances) limits the extent to which we can assess or estimate latent (psychological) processes that may be underlying the decision/behavior. To address these limitations, *cognitive process models* were developed, which use theoretically derived model parameters that represent latent psychological constructs to better account for individual differences in the complex processes underlying human decisions and behavior; see, for example, Stout et al. (1), Yechiam et al. (2), Cohen et al. (3), and Hauser et al. (4) for a variety of models and applications.

In this paper, we focus on one particular process model, the Linear Approach to Threshold with Ergodic Rate (LATER) model, which was developed to capture individual differences in the underlying mechanisms of decision-making using data from reaction time tasks (5, 6). We extend the basic LATER model hierarchically in order to assess sources of both individual and experimental condition specific differences in reaction times. Moreover, we cast the hierarchical LATER model in the Bayesian framework, which provides a convenient approach for simultaneous estimation of person-specific LATER process parameters and regression coefficients related to person-specific (e.g., age) and condition-specific (e.g., experimental manipulation of reward, smoking status) effects. Additionally, casting the model in the Bayesian framework allows for inference in terms of statements about posterior probabilities. We assert that coalescing advanced process models with experimental manipulations (e.g., abstinence vs. smoking to satiety in smokers) can help us better understand how drug exposure (e.g., nicotine) affects the underlying mental processes guiding decision-making and behavior, and may provide insights for a better understanding of addiction, particularly at the individual level.

In the sections that follow, we first describe the use of process models and specify the LATER model we hierarchically extended and employed. We then apply this novel model to reaction time data obtained from a sample of adult daily smokers to demonstrate its potential utility in addiction research.

### Modeling Reaction Times with the LATER Model

The time interval between stimulus presentation and initiation of a behavioral response is defined as the reaction time, or latency, and includes multiple underlying physiological processes occurring on varying time scales. For example, relatively rapid processes, on the order of tens of milliseconds, include transduction of the external stimulus energy to a neural response, signal propagation time from the periphery to the central nervous system and back, and muscle activation, among others. More temporally extended processes comprising reaction time include brain network-level computations (on the order of hundreds of milliseconds) related to making a decision, that is, forming and maintaining internal representations of the stimuli, then planning and executing a goal-directed motor plan. It is believed that these central, network-level computations comprise a majority of the reaction time (7, 8). As fast sensory and motor times are relatively fixed, reaction time variability is therefore a useful approximation of *decision time* (9). In other words, reaction time largely reflects the time needed to decide.

Researchers utilize tailored tasks that attempt to delineate the cognitive processes underlying reaction times in order to gain insight into decision processes and factors that influence them. However, reaction times are typically evaluated in terms of average performance across groups and/or study conditions. This approach disregards the potential variability in the processes underlying latency values, i.e., intraindividual variability across trials in a task. Indeed, in experimental paradigms, reaction time can vary significantly between one trial to the next, even if the same experimental conditions are maintained (9).

Capturing variability in reaction times with process models can provide additional information about the underlying mechanisms of decisions. One major theoretical framework for understanding decision-making holds that the brain accumulates relevant information until the resultant probability reaches a threshold that warrants action (10). The length of time in which it takes to reach this threshold depends on the dynamics of the rise-to-threshold (10). The LATER model describes the latency distributions of observed reaction times by characterizing the decision-making process in terms of two cognitive variables. The first is *caution*, or the amount of information needed to exceed a threshold to respond. The caution parameter represents the attitude toward partial prior information in a similar manner as a loss function represents the attitude toward risk (11). The second variable is *accretion*, or the rate (speed) of information accumulation. Bickel and colleagues (11) argue that caution can be seen as assigning an operational definition to the degree of conservatism toward ambiguity, and accretion rate as the assimilating capacity.

Utilizing the LATER model to describe reaction time data based on accretion rates and caution thresholds better reflects the actual shape of reaction time data relative to traditional averaging approaches. One of the most salient properties of the stochastic distribution of reaction times is that they are generally positive skewed; the distributions rise rapidly and then fall off slowly with a long, right-tailed skew. This is a near universal finding, regardless of stimulus type (e.g., visual, auditory), response (e.g., manual, oculomotor), or species [see Ref. (12)]. Interestingly, when plotted, this skewed distribution does not fit any of the traditional mathematical distributions like Gaussian or Poisson particularly well [e.g., Refs. (9, 12, 13)]. However, if one wants to examine the underlying mechanisms for the variability, rather than its effect (14, 15), then the *reciprocal* of the reaction time should be examined. If reciprocal latencies are plotted cumulatively (a reciprobit plot), a straight line will be obtained. This represents the *rate* at which the decision reaches completion, and follows a normal, Gaussian distribution (see below). Accordingly, the LATER model explains this general feature of reaction time distribution by appropriately modeling the rate of rise for each trial, varying in a Gaussian fashion, which explains the observed shape of latency distributions [see Ref. (12) for review].

This results in describing reaction time distributions by utilizing a model with a decision signal starting point, which then rises at a constant rate until it reaches a threshold value, at which point a response is initiated. Accordingly, the LATER model is a sequential-sampling model, which assumes that during the course of a trial, information is accumulated sequentially until a threshold amount of information is reached and a response is executed. Indeed, the LATER model explains the observed features of reaction time distributions by assuming that a stimulus triggers a neuronal decision signal to rise linearly until it reaches a threshold value in which a response is then executed. This rate of rise for each trial varies in a Gaussian fashion, explaining the observed shape of latency distributions. Modeling reaction time with the LATER model has provided novel insight into the cognitive components (accretion, caution) underlying reaction times in healthy individuals [see Ref. (12) for review and additional details on the original LATER model].

We argue that the LATER model can benefit from being cast in a hierarchical/multilevel framework (16, 17). Oravecz et al. (18) described a hierarchical extension to the LATER model that allowed for a person-specific accretion rate. We extend this approach by allowing for individual differences in both accretion *and* caution parameters. The multilevel extension enables us to model the individual-level repeated measures of reaction times with the LATER process and pool information across the resulting latent, person-specific accretion and caution parameters *via* joint population (group-level) distributions. The multilevel framework also provides us with a statistically principled way to add person-level predictors on these two latent parameters (e.g., to test if the number of cigarettes smoked per day is related to slower information accumulation). In our proposed model, all latent person-specific parameters and corresponding regression coefficients are estimated simultaneously, as opposed to first obtaining point estimates of caution and accretion for each person and then regressing those on predictors, which can lead to bias in the regression coefficient estimates [see Ref. (19)]. Importantly, we will also introduce condition-specific predictors to capture how accretion and caution differ as a function of experimental manipulation (e.g., smoking as usual vs. abstinence). The estimation of condition and person-specific effects is again simultaneous. The ability to have different groups and experimental manipulations within the same model also allows for direct statistical comparisons between the conditions/groups.

### Specification of the Hierarchical LATER Model

Next we introduce the model specification for the hierarchical LATER model. We start with describing the LATER model as originally outlined [see Ref. (5); for reviews see Refs. (9, 12)], but with multilevel extensions to both caution and accretion parameters. Then we describe how the single-step regression is formulated on the person-specific caution (threshold) and accretion rate (information accumulation), and we finish with showing how condition-specific effects can be incorporated in the same model.

Data will be denoted as *y* *^p,i^* for person *p* and trial *i*. We allow each subject *p* to have their own accretion (*v* *_p_*) and caution (ခθ*_p_*) parameters. On a trial *i*, a trial and person-specific realization of the accretion rate, *z* *_p,i_* is modeled through a normal (Gaussian) distribution with the following specification:

(1) $$Z_{p,i}\sim N(v_{p},1)$$

We can get the predicted response time (or latency) at trial *i* for person *p* (*y* *_p,i_*) by dividing the person-specific caution by the person-specific accretion rate on trial *i*:

$$y_{p,i}=\frac{\theta_{p}}{Z_{p,i}},$$

which can be rearranged to yield:

$$\frac{z_{p,i}}{\theta_{p}}=\frac{1}{y_{p,i}}$$

To get the distribution of $\frac{z_{p,i}}{\theta_{p}}$, we divide the distribution of *z* *_p,i_* specified in Equation 1 by θ*_p_*:

$$\frac{z_{p,i}}{\theta_{p}}=\frac{1}{y_{p,i}}\sim N\left(\frac{v_{p}}{\theta_{p}},\frac{1}{\theta_{p}^{2}}\right)$$

**Figure 1:**
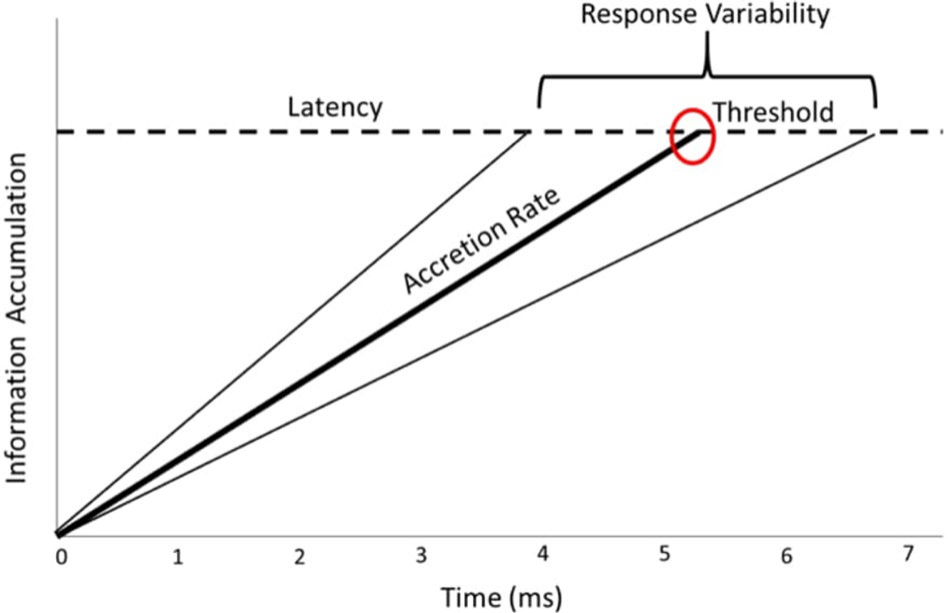
Visual representation of the cognitive processes (accretion rate and caution threshold) examined in the Linear Approach to Threshold with Ergodic Rate (LATER) model.

To summarize, the LATER model assumes sequential sampling; it assumes that over the course of a trial, information is accumulated sequentially until a threshold amount of information is reached, at which time a response is executed. This resulting accretion process (i.e., information accumulation) is assumed to be linear and eventually reaches a fixed threshold, with a rate that is random from trial to trial, as shown in **Figure 1**. Importantly, this trial-to-trial random rate is one of the key motivations to model reaction time with the LATER model approach.

To model similarities across individuals in terms of accretion and caution, we will assume that all person-specific LATER process parameters come from joint group-level (or level-2 or population) distributions. These group-level distributions also provide for a straightforward manner to regress these parameters on relevant person predictors (e.g., cigarettes smoked per day) to further improve the model. Therefore, in our application, the means of the population distributions of caution and accretion are made into the function of person predictors. Assume that *K* person covariates are measured and x*_p,k_* denotes the score of person *p* on covariate *k* *(k* = 1, *…*, *K)*. For example, in our application we considered that age, gender, cigarettes smoked per day, and nicotine dependence level (as assessed by the Fagerström Test for Nicotine Dependence; FTND) could be possible sources of individual differences among persons; therefore, we included them as person predictors. All person-specific covariate scores are collected into a vector, with the length *K*+1, denoted as **x** *_p_* = (1, x*_p_*_1_, x*_p_*_2_, …, x*_pK_*)^T^, where the first element is an intercept. The group-level distribution of the person-specific accretion parameters ν*_p_* is then formulated as

$$v_{p}=x_{p}\beta v+\varepsilon_{p,v}$$

where vector **β**_ν_, of dimension 1 × (K+1), contains the regression weights for the person predictors (e.g., association between FTND and accretion) and ε*_p,v_* is normally distributed with mean 0 and variance $\sigma_{v}^{2}$, quantifying residual unexplained inter-individual differences (random effects). Following similar logic, the group-level distribution of the person-specific caution parameters was modeled similarly: θ*_p_* = **x** *_p_* β_θ_ + ε*_p,_*_θ_. Besides person-specific differences, covariates capturing experimental conditions can also be included in the model. In our application (described below), smokers completed the task under two conditions, smoking as usual vs. abstinent. Abstinence was operationalized as abstaining from smoking for a minimum of 12 h. Baseline measures of exhaled CO were taken during a screening procedure, allowing for verification of an abstinence state. The task was composed of two trial types, reward vs. neutral. The design was completely crossed; all participants completed both conditions and trial types (smoking as usual-reward trials, smoking as usual-neutral trials, abstinent-reward trials, abstinent-neutral trials). We selected smoking as usual and neutral as the baseline, and dummy coded the neutral–abstinent, reward–abstinent, and reward–smoking as usual conditions. The regression coefficients corresponding to these dummy-coded condition-specific variables represent the deviations of a condition from the baseline (i.e., smoking as usual-neutral reward).

We denote these covariates for every data point as *g* *_n,c_* where *n* = (1, 2, …, *N*), with *N* representing the total number of reaction times in the experiment and *c* = (1, 2, …, *C*), and *C* representing the number of dummy-coded conditions minus 1 (baseline). Corresponding regression coefficients are denoted as δ*_v,c_* for accretion and δ_θ_ *_,c_* for the caution threshold. **Table 1** shows the conditions (reward vs. neutral and smoking as usual vs. abstinent) with corresponding regression terms for further clarification of the design. To formulate the LATER model with these experimental condition effects, we introduce a more general notation than that of Equation 1 for data *y* *_p,i_*: we stack all trials for the persons *p* under each other, resulting in a long vector of reaction time scores, where *n* stands for a single trial (up to *N*), and then we rewrite the model as:

$$y_{n}\sim N\left(\frac{v_{n}}{\theta_{n}},\frac{1}{\theta_{n}^{2}}\right)$$

**Table 1:** Describes the design matrix of the current study; the two conditions (Smoke as Usual, Abstinent) and two trial types (reward, neutral) with corresponding regression terms are shown here for the person-specific pater parameters.

	Smoke as Usual	Abstinent
Neutral	δ*_v_*_,1_,δ_θ,1_	Baselines
Reward	δ*_v_*_,3_,δ_θ,3_	δ*_v_*_,2_,δ_θ,2_

For example, with the three conditions we introduced, the accretion is then modeled as:

$$v_{n}=v_{p}+g_{n,}{}_{1}\delta_{v,}{}_{1}+g_{n,}{}_{2}\delta_{v,}{}_{2}+g_{n,}{}_{3}\delta_{v,}{}_{3}$$

which can be written in a more general form:

$$v_{n}=v_{p}+g\delta_{v}$$

Similar formulation applies to the caution parameter:

$$\theta_{n}=\theta_{p+}g\delta_{\theta }$$

This formulation allows us to model the effect of the experimental manipulation in terms of meaningful process model parameters while also capturing individual differences in these parameters.

### Modeling in the Hierarchical Bayesian Framework

The hierarchically extended LATER model was cast in the Bayesian framework. In this framework, both data and model parameters are defined as random variables and the Bayesian model specifies their joint probability distribution (20). With this approach, statistical inference is focused on the posterior probability distribution of the parameters, which is derived by combining the likelihood and prior distribution on the model parameters based on Bayes’ rule. The prior distributions are integral parts of the model; the mean of the prior suggests the likely parameter value, and the variance of the prior distribution reflects the level of uncertainty about the possible values of the parameter of interest. This analysis is the mathematically normative way to reallocate credibility across parameter values as new data arrive (21).

In the Bayesian framework, inferences about parameters are based on the posterior probability distributions of the parameters. The posterior distribution is stochastically approximated by taking a large number of samples from it, and then calculating posterior point estimates, posterior standard deviations (similar to that of the standard error), and posterior credible intervals for each parameter. One of the key strengths in fitting a hierarchical model with a Bayesian statistical approach is that these algorithms are able to fit increasingly complex models to the data (22). This is especially useful for our model as we can estimate all person-specific parameters, group-level variances, and regression coefficients corresponding to person and condition effects simultaneously. Parameter estimation was implemented in Stan (23); software code for the model is provided in **Appendix A**. The utilized data and accompanying R script are also provided as an **Online Supplement** on the project’s Open Science Framework (OSF) page: https://osf.io/5h8m4/?view_only=f6c1e50dcfa04244bba428d6cf259d36

### Model Application—Smokers

We fit the hierarchical LATER model to data from “go” trials from a Go/No-Go task performed by a group of adult daily smokers to gain further insight into cognitive changes associated with smoking abstinence. While the Go/No-Go task is a paradigm typically used to investigate inhibitory control (no-go trials), it can also be a highly informative task in terms of assessing what cognitive mechanisms support “go” decisions (12, 24). Notably, go trials in this task far outnumber the number of no-go trials, increasing power and adding an additional dimension of rich data to analyze from this classic task. Prior studies have utilized the Go/No-Go behavioral paradigm to study the effect of nicotine use on cognitive systems using reaction times [e.g., Refs. (25–27)]; these studies manipulate the task environment in various ways, such as smoking status (e.g., daily smoker vs. non-smoker) and session type (e.g., smoking to satiety vs. abstinent). However, findings from these studies thus far have only demonstrated differences in reaction times (and error rates) between these various manipulations. While these studies have been informative in highlighting the fact that nicotine impacts task performance under particular task manipulations, they fail to explain how. That is, what are the underlying mechanisms of reaction times (i.e., components of decision-making) that nicotine affects?

Given widespread effects of nicotine on cognitive brain systems [e.g., Refs. (25, 26, 28–35)], we hypothesize that nicotine will affect psychological (cognitive) processes important for decision-making, including caution threshold and accretion rates. Furthermore, given that nicotine is known to alter (decrease) responsiveness to non-drug (e.g., money), particularly during periods of smoking abstinence [e.g., Refs. (33, 36–38)], we hypothesize that the availability of rewards may differentially impact caution and accretion depending on smoking status, as these likely interact with reward processes during incentivized decision-making [e.g., Ref. (29)]. We suggest that these effects may be masked or confounded when analyzing latencies via traditional average mean scores. In addition, traditional analysis is often based on averaging task performance across individuals per experimental condition, disregarding possible intraindividual differences that may be present. Failure to account for such differences may contribute to inconsistent results found in previous work [see Ref. (39)]. By utilizing the LATER process modeling approach instead of relying on statistical summaries of raw reaction times, substantively meaningful latent model parameters (accretion and threshold) are calculated and updated in a trial-by-trial manner, better capturing intraindividual processes. Moreover, by allowing individual differences in the latent process model parameters, this ensures that condition-specific differences are not biased by an averaging artifact. To this end, our proposed modeling approach was employed in an attempt to elucidate the effects of nicotine exposure (smoke as usual vs. abstinence) on cognitive functioning and potential moderating effects of rewards on Go/No-Go task performance.

The current dataset has previously been explored via the traditional frequentist approach to examine the effects of reward and smoking conditions on the latency and accuracy of task performance (see Ref. 40). However, it is not well understood which cognitive parameters nicotine affects. As a result, it remains unknown if non-drug rewards affect particular components of cognitive functioning in smokers. One goal in extending the LATER model was to explore intraindividual differences among daily cigarette smokers in their information accumulation and caution cognitive processes. In addition, we also wanted to study the difference in these two processes across experimental conditions (i.e., reward/neutral condition; smoke as usual/abstinence).

## Methods

### Participants

After Institutional Review Board approval, 23 smokers were recruited *via* community advertisements. Inclusion criteria were the following: a) ≥18 years old, b) smoked at least four cigarettes/day for the past 12 months, c) inhale while smoking, and d) no intention to quit smoking in the next 1 month. Exclusion criteria were the following: a) women who were pregnant or lactating, or who planned to become pregnant or breastfeed during the study, and b) other tobacco use within the past 12 months. Participants who dropped out before completing the study (*n* = 5) were excluded, leaving a final sample of 17 (5 females). While this is a relatively low sample size, each person has a high number of trials (750), which facilitate the estimation of the person-specific process parameters. Fewer trials would certainly result in more uncertainty (higher posterior standard deviation) in the parameter estimates; however, via hierarchical modeling, we pool information across participants to improve parameter estimation. Moreover, a large number of trials in fact are not uncommon in the Go/NoGo literature, as it helps build a prepotent response. In addition, as we take a multilevel modeling approach, we pool information across persons, which helps handle outlier effects and reduces the risk of model over-fitting. The mean age of these participants was 31.06 (*SD* = 13.82). Participants identified as Caucasian, (66.7%), Asian (27.8%), and mixed race (5.6%). Participants reported smoking an average of 11.08 cigarettes per day. The sample exhibited low nicotine dependence on the Fagerström Test of Nicotine Dependence (FTND), with a mean score of 2.61 (*SD* = 2.35).

### Procedure

Participants attended a baseline session. A coVita|Bedfont Micro Smokerlyzer^®^ was used to monitor CO levels. The Beck Depression Inventory–II (41) and the Center for Epidemiologic Studies Depression Scale–Revised (42) were used to screen for current depression. A screening for dependence on drugs other than nicotine was also administered. Participants then completed the FTND (43). Participants then attended two counterbalanced sessions—smoke as usual and abstinent. For the abstinent session, participants were instructed not to smoke for at least 12 h before the session. For the smoke as usual session, participants were instructed to continue their regular smoking habits.

Participants began the experimental sessions by providing a CO sample to ensure abstinence or smoke as usual conditions. Abstinence was determined by a CO level of at least one half of the participant’s CO level at their baseline session. Individuals then completed a recent nicotine, alcohol, and substance use measure, and the Questionnaire of Smoking Urges–Brief (QSU) (44). Participants reporting the use of alcohol or other substances within 24 h before experimental sessions were asked to return at a later date when they had refrained from substance use. Investigators then administered a measure of nicotine withdrawal, followed by an antisaccade (inhibitory control) and a working memory task (not reported here), as well as a monetary incentivized Go/No-Go task. Each session lasted approximately 2 h. Results of questionnaires utilized in the current analyses and additional demographics can be found in **Table 2**.

**Table 2:** Participant characteristics.

	Mean	SD
Age	34.15	18.31
Age of first use	19.63	5.34
FTND	2.63	11.32
Avg. cigarettes per day	2.29	11.00

FTND, Fagerström test for nicotine dependence.

### Go/No-Go Task

An incentivized version of the Go/No-Go task was administered *via* a computer with a 17-in. monitor presented in E-Prime (Psychology Software Tools, Inc., Pittsburgh, PA). The task consisted of three trial types: frequent-Go (FGO; 75%), infrequent-Go (IFGO; 12.5%), and NoGo trials (12.5%) (45). Only data from the FGO trials are analyzed in this study as a main aim of the current modeling approach was to examine inter-individual variability in reaction times. Including IFGO trials would introduce additional sources of variability, confounding the findings. The participants were required to press the space bar on a computer keyboard using the index finger of their dominant hand. Each trial consisted of the presentation of a colored square for 400 ms followed by the presentation of a fixation cross for 400 ms. Responses were collected during this 800-ms period. Participants were instructed to respond as quickly and as accurately as possible. Trials with reactions times <150 ms were excluded from analyses to avoid the inclusion of potentially premature responses. This was a threshold that we set in order to ensure that the response was in fact a reaction to the stimulus. If reaction times are too fast, they are not a reaction to the stimulus; rather they reflect general responding. Utilizing a threshold is well documented in the reaction time literature [see, e.g., Ref. (40)] (46). The trial types were presented pseudo-randomly. Participants completed 10 runs, and each run was composed of 100 trials. Five runs were preceded by a ring of dollar signs ($), indicating the availability of monetary reward depending on run performance. Five runs were preceded by a ring of pound signs (#), indicating that no monetary reward was available. The order of runs was randomized. Participants were instructed that they could earn up to $5.00 in addition to their participation earnings, and that faster and more accurate performance on rewarded blocks would result in a greater reward amount. Participants were instructed that they would receive the earned rewards once they had completed the study and the investigators analyzed their data. At the end of the trials, the participants were told that they were getting the full reward amount.

### Bayesian Data Analysis

In the present application of the model, we used weakly informative prior distributions, specified in **Appendix A**. As we had no prior knowledge, we chose weakly informative priors so that the prior distributions would have very little impact on the results. Parameters were estimated by running six chains with 2,000 iterations each, discarding the first 1,000 samples as burn-in. Convergence of the six chains was tested by the $\hat{R}$ statistic (the Gelman–Rubin convergence statistic, used to test the degree of convergence of a random Markov Chain; see Ref. 47). $\hat{R}$ is calculated by taking the ratio of variance within and between chains. $\hat{R}$ was lower than 1.01 for all parameters (conventional criterion being $\hat{R}$ <1.1), indicating no problems with convergence. The full R script and accompanying data that allow for replicating the analysis can be found on the Open Science Framework website of the project^1^.

## Results

### Individual Differences in the Decisions on Go Trials

**Figure 2:**
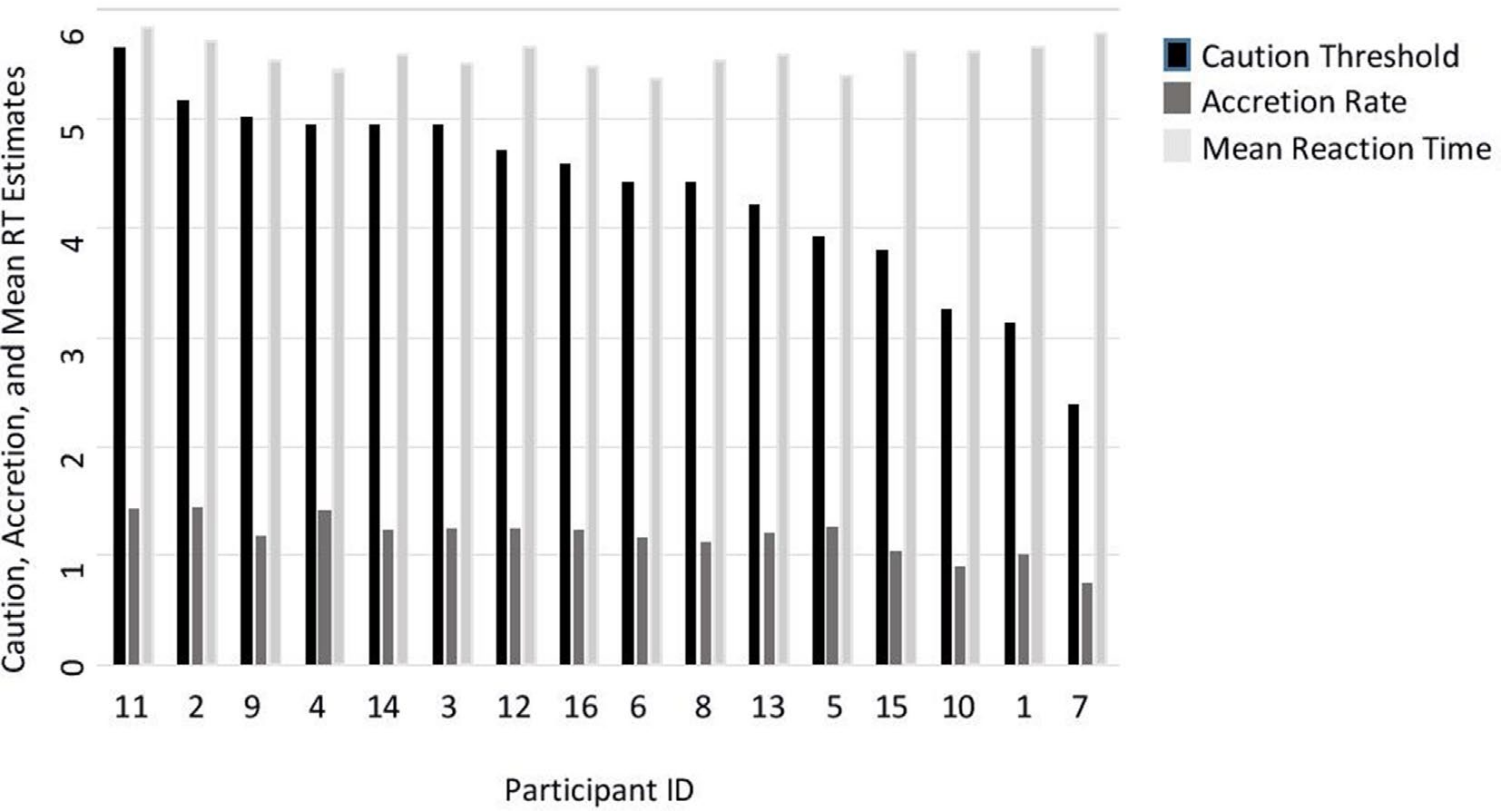
Individual differences in participants’ accretion, caution, and mean reaction time (RT) estimates. *Note*. Mean RT is log transformed. Mean reaction times were included in the figure to demonstrate the different combinations of caution and accretion, which could result in similar RTs.

We estimated an accretion and a caution threshold parameter for each person. Results show individual differences in accretion rate and caution threshold (**Figure 2**). Caution parameter estimates ranged between 2 and 6, while accretion rate was between 0.7 and 1.6. To relate these two scales, a person, for example, with caution parameter 4 and accretion rate 1 would need ¼ s (250 ms) to give a response. Alternatively, the same reaction time can arise from a faster accretion rate (e.g., 1.5) but also higher caution (e.g., 6). As can be seen in **Figure 2**, various combinations of accretion rates and caution parameters can result in very similar reaction times.

We included person-level predictors (chronological age, age of smoking initiation, FTND score, average number of cigarettes smoked per day) to predict individual differences in accretion or caution, but no predictors explained differences in either parameter. Regression coefficients estimates and corresponding 95% credible intervals are reported in **Appendix B**.

### Condition-Specific Differences in the Decisions on the Go Trials

We were interested in capturing differences in the decisions on the Go trials in periods when smokers abstained from smoking (vs. smoking as usual) and when a reward was offered depending on their performance (vs. neutral condition with no reward). These conditions were crossed for each person for a two-by-two design. We chose the neutral trials in the smoke as usual session as our baseline, and modeled the differences in the neutral and abstained from smoking, and the abstinent and smoking as usual reward conditions in terms of accretion and caution. Results are reported in **Table 3**. All accretion parameters had posterior distributions that had posterior mass largely concentrated away from zero, indicating support for a difference in these conditions on accretion, compared to the baseline (neutral trial, smoke as usual) condition. The δ*_v,1_* and δ*_v,3_* accretion parameters reveal that regardless of trial condition (neutral vs. reward), abstaining from smoking was associated with faster information accumulation compared to smoking as usual. The δ*_v,2_* accretion estimate indicated that when smoking as usual, smokers had slower accretion rates relative to reward trials.

**Table 3:** Summary of the regression weights where response speed was modeled with the LATER model.

Condition	Posterior mean	Posterior SD	95% CrI
Neutral, abstinent	−0.3638	0.0552	(−0.4763, −0.2613)
Reward, smoke as usual	0.1231	0.0565	(0.0153 , 0.2360)
Reward, abstinent	−0.2494	0.0556	(−0.3573, −0.1384)
Neutral, abstinent	−0.0655	0.0145	(−0.0938, −0.0376)
Reward, smoke as usual	0.0835	0.0150	(0.0537, 0.1120)
Reward, abstinent	−0.0068	0.0147	(−0.0352 , 0.0222)

Negative Posterior Means indicate faster accretion rates and lower caution thresholds; positive values indicate slower accretion rates and higher caution thresholds. Mean and SD are posterior mean and standard deviation. “Neutral” refers to a neutral trial; “Reward” refers to a reward trial. CrI, credibility interval; SD, standard deviation.

Compared to the baseline condition, regardless of trial condition, smokers had a lower caution threshold when in a period of abstinence, relative to the baseline condition (◍δ_θ,1_,δ_θ,3_) The δ_θ,3_ parameter had a 95% confidence interval containing 0, indicating less confidence for a meaningful difference between this parameter (abstinent, reward) to the baseline (smoke as usual, reward). The δ_θ,2_ parameter indicates a larger caution threshold in the reward trials relative to the neutral trials in the smoke as usual session, suggesting that participants are integrating reward information into their cognitive appraisals of whether or not to execute a “go” response.

### Model Fit

**Figure 3:**
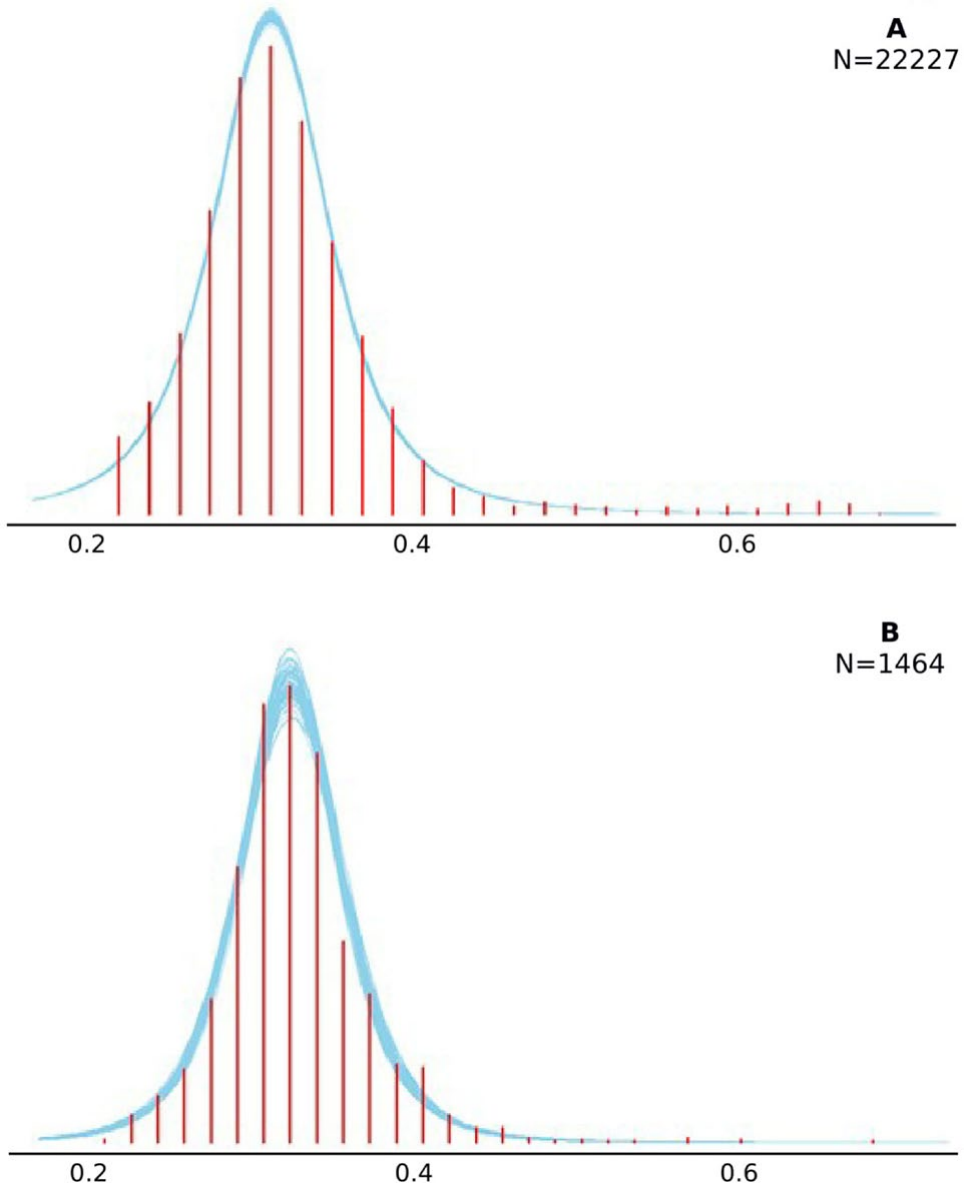
Visual summary of the posterior predictive checks. Checks were completed with 100 generated data sets. Smoothed histograms of these generated datasets are depicted by the blue curves. The distribution of the experimental data is shown with the red bars. Plot **(A)** shows these checks on the level of the full data set, while Plot **(B)** shows it for a randomly selected participant.

In addition to overall model convergence, we tested how well the LATER model fit the actual observed data through posterior predictive checks (PPCs). For this, we generated 100 new data sets from the posterior distributions of the LATER model parameters. **Figure 3** shows smoothed blue curves of these generated datasets overlaying the experimental data (red histogram). Plot A depicts the full data PPC results, and Plot B displays a randomly selected participant’s data. Overall, the LATER model adequately fit the experimental data well, demonstrated by generated data sets, which nicely overlay the real experimental data (i.e., the blue curves follow the same pattern of the red histogram). The results were analyzed in the Bayesian framework, which does not utilize traditional indices to show goodness of fit (e.g., CFI) but relies on PPCs. This entails “simulating replicated data under the fitted model and then comparing these to the observed data” (48, p. 158). Systematic discrepancies within these graphical checks are indicative of poor model fit. Here, our graphical PPCs shown in **Figure 3** do not reveal any systematic misfit.

## Discussion

In this paper, we articulated model implementation of a novel hierarchically extended LATER model, which parses reaction time into two distinct aspects of cognitive functioning: accretion rate and caution threshold. This model extension enables researchers to account for and compare differences in sources of variation related to experimental conditions and person-specific differences in accretion and threshold. We demonstrated the applicability and benefits of this model by applying it to reaction time data from a group of adult daily smokers, identifying condition and trial level effects. We aimed to place emphasis on both modeling and the nuanced substantive findings that this modeling makes possible. That is, we presented a novel hierarchical extension to the LATER model in order to account for differences across persons and experimental conditions simultaneously. We showcase the strength of this approach by demonstrating what researchers can learn about smoking status and the influence of rewards utilizing this modeling approach.

In the original analyses of the data, Lydon et al., (40) reported that task performance was more accurate (in regards to error processing) on rewarded trials relative to the neutral trials, but only in the smoke as usual session. There were no differences between reward and neutral trials during the abstinent session. And importantly, there were no significant differences in mean reaction times between the abstinent and smoke as usual sessions, regardless of the trial type. Here, our findings demonstrate differences in both cognitive parameters underlying reaction times.

In the current analyses, in the accretion parameter, the baseline (or comparative) condition was smoke as usual, neutral trials. Our results demonstrated the following: Relative to our baseline condition, when smokers were in an abstinent state, they had faster accretion rates in both reward and neutral trials. When smokers were smoking as usual, they had slower accretion rates when a reward was at stake relative to neutral trials. In regards to the caution threshold, again the baseline was smoke as usual, neutral trials. Relative to this baseline condition, when a participant was in a period of abstinence, regardless of the trial type (reward, neutral), s/he utilized a lower caution threshold. Compared to the baseline smoke as usual neutral condition, when a reward was at stake (still smoking as usual condition), smokers utilized a larger caution threshold.

Our study is the first to combine advanced process models with experimental manipulations to examine the effects of smoking on behavior. Understanding how rewards affect decisions is critical as contingency management treatment programs encourage continued abstinence by increasing the value associated with continued abstinence (49). Our findings demonstrate differences in both accretion and caution parameters when smokers were abstinent relative to smoking as usual: faster accretion rates and lower caution thresholds when participants were in a period of abstinence, regardless of trial type. This overall main finding falls in line with other studies demonstrating abstinence-related reward-insensitivities (28, 33, 36), with important implications for contingency management programs. If incentives used in smoking interventions are not overcoming cognitive deficits produced by acute nicotine withdrawal, incentives may fail to change the value associated with continued smoking abstinence, undermining the allocations of cognitive resources needed in attempts to remain abstinent. Future work should focus on examining the generalizability of reward/reward insensitivity, particularly in an abstinence state, to other types of motivating incentives (e.g., food, social praise) in order to investigate if alternative incentives can impact cognitive performance in deprived smokers in order to inform the development of effective interventions.

Interestingly, when smokers were smoking as usual, rewarded trials produced slower accretion rates and increased caution thresholds. This finding suggests that when participants were smoking as usual, they seemed to be more careful in their decision time, perhaps a speed-accuracy tradeoff. Indeed, Lydon et al. (40) reported fewer errors when examining the no/go trials of this task in rewarded vs. neutral trials when participants were smoking as usual. Additionally, additional processing demands/time could have been needed in order to integrate information about the reward into the decision process.

To our knowledge, a LATER process model has never been applied to cigarette smokers to examine the underlying mechanisms of reaction or decision times. However, our findings fall in line with other research groups attempting to examine differences in underlying mechanisms of decision-making based on smoking state. Zack et al. (50) found that adolescent heavy smokers made more errors on a rapid information processing task relative to when they were smoking as usual, in line with the current results. These results support the notion that that accretion rate, the speed of information accumulation, is affected by abstinence. In a resting state magnetic resonance imaging study, Lerman and colleagues (30) reported that weaker inter-network connectivity (salience and default) predicted less suppression of default mode activity during performance of a working memory task. They argue that alterations in the coupling of these networks, and the inability to disengage from the default mode network, may be critical in cognitive alterations that underlie dependence. In our study, the trial type (reward vs. neutral) did not make a difference when smokers were in a period of abstinence. This could be due to alterations in the coupling of these networks as found in the study by Lerman and colleagues.

There are notable limitations in the current study. We implemented our model in the Bayesian statistical framework, which allowed us to fit a complex model to reaction time data in a single step. However, there are limitations to utilizing a Bayesian framework, namely in the computation power needed to implement such approaches. The current analysis was carried out using parallel computations [six cores running six Markov chain Monte Carlo (MCMC) chains] and took about 25 min. However, due to recent advances in statistical software, computational difficulty is becoming less of an issue. In addition, we had a limited sample size and unbalanced gender. However, as described in our Methods section, our implementation of a process model that utilizes a sequential sampling method and hierarchical modeling handles small sample sizes better than traditional approaches. We have made our scripts and data available to facilitate researchers utilizing this approach, hopefully with larger samples and more balanced samples to overcome this limitation in future work.

Taken together, our hierarchical extension of the LATER process model is able to separate the reaction time of the go trials into two cognitive processes, accretion and caution, while simultaneously accounting for differences in groups/session (smoke as usual vs. abstinent) and experimental condition (reward vs. neutral trials). Combing these approaches provides additional nuanced insight into nicotine’s effects on behavior.

Our model examines differences across individuals together with condition specific differences. This is an important extension of the model as it is critical for researchers to have the ability to test both between- and within-person differences in experimental conditions. Continual use of marrying cognitive process models with experimental condition manipulations will help elucidate factors that may impact decision-making in smokers, and can be extended to additional types of addiction. This modeling approach can and should be used in future research; by combining this approach with other tasks, group conditions, etc., researchers can better understand the cognitive processes underlying decision-making within particular groups. These cognitive factors have the potential to inform the development and improvement of intervention programs by understanding which cognitive mechanisms need to be targeted by interventions. Although we did not find an association between individual level predictors and accretion/caution parameters, our novel extension to the LATER model puts us in a position to assess this in the future with larger sample sizes, more diverse samples (e.g., varying levels of nicotine dependence), and other types of addiction.

## Ethics Statement

The study protocol was reviewed and approved by the Penn State Institution Review Board. All subjects gave written informed consent in accordance with the Declaration of Helsinki.

## Author Contributions

NR contributed to data acquisition, analysis, interpretation, drafting the manuscript, and study conception and design. BS contributed to data analysis and drafting the manuscript. ZO and CG contributed to study conception and design, data analysis, interpretation, and drafting the manuscript.

## Funding

NR received funding by the National Institute on Drug Abuse/Penn State Prevention & Methodology Trainee (T32DA017629-34) grant, and funding by the USDA 2011-6700-67001-30017 Childhood Obesity Prevention Training Program while working on this project. BS was supported by the Joseph and Jean Britton Fellowship through the Pennsylvania State University Center for Healthy Aging; additional support was provided through the Kligman Graduate Fellowship. Computations for this research were performed on the Pennsylvania State University’s Institute for CyberScience Advanced CyberInfrastructure (ICS-ACI). CG was supported by the Dr. Frances Keesler Graham Early Career Professorship in Developmental Neuroscience.

## Conflict of Interest Statement

The authors declare that the research was conducted in the absence of any commercial or financial relationships that could be construed as a potential conflict of interest.

## Footnotes

1 https://osf.io/5h8m4/?view_only=f6c1e50dcfa04244bba428d6cf259d36

## References

[1] Stout JC Busemeyer JR Lin A Grant SJ Bonson KR Cognitive modelling analysis of decision-making processes in cocaine abusers. Psychon Bull Rev (2004) 11(4):742–7. 10.3758/BF03196629 15581127

[2] Yechiam E Busemeyer JR Stout JC Bechara A Using cognitive models to map relations between neuropsychological disorders and human decision-making deficits. Psychol Sci (2005) 16(12):973–8. 10.1111/j.1467-9280.2005.01646.x 16313662

[3] Cohen JR Asarnow RF Sabb FW Bilder RM Bookheimer SY Knowlton BJ A unique adolescent response to reward prediction errors. Nat Neurosci (2010) 13(6):669. 10.1038/nn.2558 20473290 PMC2876211

[4] Hauser TU Iannaccone R Walitza S Brandeis D Brem S Cognitive flexibility in adolescence: neural and behavioral mechanisms of reward prediction error processing in adaptive decision making during development. Neuroimage (2015) 104:347–54. 10.1016/j.neuroimage.2014.09.018 PMC4330550 25234119

[5] Reddi BAJ Carpenter RH The influence of urgency on decision time. Nat Neurosci (2000) 3(8):827. 10.1038/77739 10903577

[6] Ratcliff R Putting noise into neurophysiological models of simple decision making. Nat Neurosci (2001) 4(4):336. 10.1038/85956 11276213

[7] Brenner E Smeets JB How people achieve their amazing temporal precision in interception. J Vis (2015) 15(3):8–8. 10.1167/15.3.8 25767094

[8] Rubinstein A Response time and decision making: an experimental study. Judgm Decis Mak (2013) 8(5):540–51.

[9] Noorani I LATER models of neural decision behavior in choice tasks. Front Integr Neurosci (2014) 8:67. 10.3389/fnint.2014.00067 25202242 PMC4141543

[10] Noorani I Carpenter RHS Re-starting a neural race: anti-saccade correction. Eur J Neurosci (2014) 39(1):159–64. 10.1111/ejn.12396 24168375

[11] Bickel WK Jarmolowicz DP Mueller ET Gatchalian KM McClure SM Are executive function and impulsivity antipodes? A conceptual reconstruction with special reference to addiction. Psychopharmacology (2012) 221(3):361–87. 10.1007/s00213-012-2689-x PMC4035182 22441659

[12] Noorani I Carpenter RHS The LATER model of reaction time and decision. Neurosci Biobehav Rev (2016) 64:229–51. 10.1016/j.neubiorev.2016.02.018 26915927

[13] Whelan R Effective analysis of reaction time data. Psychol Rec (2008) 58(3):475–82. 10.1007/BF03395630

[14] Carpenter RHS Reddi BAJ Anderson AJ A simple two-stage model predicts response time distributions. J Physiol (2009) 587(16):4051–62. 10.1113/jphysiol.2009.173955 PMC2756437 19564395

[15] Carpenter RHS Contrast, probability, and saccadic latency: evidence for independence of detection and decision. Curr Biol (2004) 14(17):1576–80. 10.1016/j.cub.2004.08.058 15341745

[16] Bryk AS Raudenbush SW Application of hierarchical linear models to assessing change. Psychol Bull (1987) 101(1):147. 10.1037//0033-2909.101.1.147

[17] Raudenbush SW Bryk AS Hierarchical linear models: applications and data analysis methods. Sage (2002) 1.

[18] Oravecz Z Huentelman M Vandekerckhove J Sequential Bayesian updating for big data. In: Big Data in Cognitive Science. London and New York: Routledge Taylor & Francis Group (2016). p. 13–33.

[19] Pagan A Econometric issues in the analysis of regressions with generated regressors. Int Econ Rev (1984) 25:221–47. 10.2307/2648877

[20] Gelman A Carlin JB Stern HS Dunson DB Vehtari A Rubin DB Bayesian Data Analysis Vol. 2 Boca Raton, FL: CRC Press (2014).

[21] Kruschke JK Aguinis H Joo H The time has come: Bayesian methods for data analysis in the organizational sciences. Organ Res Methods (2012) 15(4):722–52. 10.1177/1094428112457829

[22] Turner RM Jackson D Wei Y Thompson SG Higgins JP Predictive distributions for between-study heterogeneity and simple methods for their application in Bayesian meta-analysis. Stat Med (2015) 34(6):984–98. 10.1002/sim.6381 PMC4383649 25475839

[23] STAN Development team Stan Modeling Language: User’s Guide and Reference Manual. Version 2.11.0. http://mc-stan.org.

[24] Noorani I Gao MJ Pearson BC Carpenter RHS Predicting the timing of wrong decisions with LATER. Exp Brain Res (2011) 209(4):587–98. 10.1007/s00221-011-2587-1 21336830

[25] Spinella M Correlations between orbitofrontal dysfunction and tobacco smoking. Addict Biol (2002) 7(4):381–4. 10.1080/1355621021000005964 14578013

[26] Dinn WM Aycicegi A Harris CL Cigarette smoking in a student sample: neurocognitive and clinical correlates. Addict Behav (2004) 29(1):107–26. 10.1016/j.addbeh.2003.07.001 14667424

[27] Reynolds B Patak M Shroff P Penfold RB Melanko S Duhig AM Laboratory and self-report assessments of impulsive behavior in adolescent daily smokers and nonsmokers. Exp Clin Psychopharmacol (2007) 15(3):264. 10.1037/1064-1297.15.3.264 17563213

[28] Dawkins L Acaster S Powell JH The effects of smoking and abstinence on experience of happiness and sadness in response to positively valenced, negatively valenced, and neutral film clips. Addict Behav (2007) 32(2):425–31. 10.1016/j.addbeh.2006.05.010 16824689

[29] Geier CF Sweitzer M Denlinger R Sparacino G Donny E Abstinent adult daily smokers show reduced anticipatory but elevated saccade-related brain responses during a rewarded antisaccade task. Psychiat Res Neuroim (2014) 223(2):140–7. 10.1016/j.pscychresns.2014.04.007 PMC5428387 24914005

[30] Lerman C Gu H Loughead J Ruparel K Yang Y Stein EA Large-scale brain network coupling predicts acute nicotine abstinence effects on craving and cognitive function. JAMA Psychiatry (2014) 71(5):523–30. 10.1001/jamapsychiatry.2013.4091 PMC4097018 24622915

[31] Perkins KA Lerman C Coddington SB Jetton C Karelitz JL Scott JA Initial nicotine sensitivity in humans as a function of impulsivity. Psychopharmacology (2008) 200(4):529–44. 10.1007/s00213-008-1231-7 18604520

[32] Sweitzer MM Donny EC Dierker LC Flory JD Manuck SB Delay discounting and smoking: association with the Fagerström test for nicotine dependence but not cigarettes smoked per day. Nicotine Tob Res (2008) 10(10):1571–5. 10.1080/14622200802323274 18946776

[33] Sweitzer MM Geier CF Joel DL McGurrin P Denlinger RL Forbes EE Dissociated effects of anticipating smoking versus monetary reward in the caudate as a function of smoking abstinence. Biol Psychiatry (2014) 76(9):681–8. 10.1016/j.biopsych.2013.11.013 PMC4026339 24342923

[34] Wilson SJ Sayette MA Neuroimaging craving: urge intensity matters. Addiction (2015) 110(2):195–203. 10.1111/add.12676 25073979 PMC4410051

[35] Zelle SL Gates KM Fiez JA Sayette MA Wilson SJ The first day is always the hardest: functional connectivity during cue exposure and the ability to resist smoking in the initial hours of a quit attempt. NeuroImage (2017) 151:24–32. 10.1016/j.neuroimage.2016.03.015 26975550 PMC5018416

[36] Dawkins L Powell JH West R Powell J Pickering A A double-blind placebo controlled experimental study of nicotine: I—effects on incentive motivation. Psychopharmacology (2006) 189(3):355–67. 10.1007/s00213-006-0588-8 17047930

[37] Powell J Tait S Lessiter J Cigarette smoking and attention to signals of reward and threat in the Stroop paradigm. Addiction (2002) 97(9):1163–70. 10.1046/j.1360-0443.2002.00117.x 12199832

[38] Peters J Bromberg U Schneider S Brassen S Menz M Banaschewski T Lower ventral striatal activation during reward anticipation in adolescent smokers. Am J Psychiatry (2011) 168(5):540–9. 10.1176/appi.ajp.2010.10071024 21362742

[39] Lee MD How cognitive modeling can benefit from hierarchical Bayesian models. J Math Psychol (2011) 55(1):1–7. 10.1016/j.jmp.2010.08.013

[40] Lydon DM Roberts NJ Geier CF Reduced influence of monetary incentives on Go/NoGo performance during smoking abstinence. Nicotine Tob Res (2014) 17(9):1178–81. 10.1093/ntr/ntu283 PMC4542739 25542919

[41] Beck AT Steer RA Brown GK Beck depression inventory–II. San Antonio (1996) 78(2):490–8. 10.1037/t00742-000

[42] Eaton WW Smith C Ybarra M Muntaner C Tien A Center for Epidemiologic studies depression scale: review and revision (CESD and CESD-R). (2004). 10.1037/t29280-000

[43] Heatherton TF Kozlowski LT Frecker RC Fagerstrom KO The Fagerström test for nicotine dependence: a revision of the Fagerström tolerance questionnaire. Br J Addict (1991) 86(9):1119–27. 10.1111/j.1360-0443.1991.tb01879.x 1932883

[44] Cox LS Tiffany ST Christen AG Evaluation of the brief questionnaire of smoking urges (QSU-brief) in laboratory and clinical settings. Nicotine Tob Res (2001) 3(1):7–16. 10.1080/14622200020032051 11260806

[45] Chikazoe J Jimura K Hirose S Yamashita KI Miyashita Y Konishi S Preparation to inhibit a response complements response inhibition during performance of a stop-signal task. J Neurosci (2009) 29(50):15870–7. 10.1523/JNEUROSCI.3645-09.2009 PMC6666181 20016103

[46] Hallett PE Primary and secondary saccades to goals defined by instructions. Vision research, 18(10) (1978) 1279–96. 10.1016/0042-6989(78)90218-3 726270

[47] Gelman A Rubin DB Inference from iterative simulation using multiple sequences (with Discussion). Statistical Science (1992) 7:457–511.

[48] Gelman A Hill J Data analysis using regression and multilevel/hierarchical models. Cambridge University Press (2006).

[49] Prendergast M Podus D Finney J Greenwell L Roll J Contingency management for treatment of substance use disorders: a meta-analysis. Addiction (2006) 101(11):1546–60. 10.1111/j.1360-0443.2006.01581.x 17034434

[50] Zack M Belsito L Scher R Eissenberg T Corrigall WA Effects of abstinence and smoking on information processing in adolescent smokers. Psychopharmacology (2001) 153(2):249–57. 10.1007/s002130000552 11205427

