# Supplementary material for: A Novel Hierarchical LATER Process Model: Evaluating Latent Sources of Variation in Reaction Times of Adult Daily Smokers
Source: Front Psychiatry. 2019 Jul 5;10:474. doi: 10.3389/fpsyt.2019.00474 (PMC6624441; doi:10.3389/fpsyt.2019.00474)
Supplement: Supplementary file 1 [file DataSheet_1.docx]

**Appendix A**

**Stan code for fitting the LATER model**

data {

int<lower=1> P; // number of participants

int<lower=1> N; // total number of datapoints

int<lower=1> K; // number of person predictors

int<lower=1> Tr; // number of condition predictors

int<lower=1,upper=P> personInd[N]; // person index for all the datapoints

vector[N] Y; // all RT data

matrix[P, K] X; // person predictor matrix

matrix[N, Tr] Xcond; // cond predictor matrix

}

parameters {

real<lower=0> sigmanu;

real<lower=0>interceptnu[P]; // information accumulation rate for person p

real<lower=0> sigmatheta;

real<lower=0> intercepttheta[P]; // caution for person p

vector[K] betanu; // regression coefficients for nu

vector[K] betatheta; // regression coefficients for theta

vector[Tr] betacondnu; // regression coefficients for condition specific effects on nu

vector[Tr] betacondtheta; // regression coefficients for condition specific effects on theta

}

transformed parameters {

real<lower=0> nu[N];

real<lower=0> theta[N];

for (n in 1:N) {

nu[n] <- interceptnu[personInd[n]] + betacondnu[1]*Xcond[n,1] + betacondnu[2]*Xcond[n,2] + betacondnu[3]*Xcond[n,3];

theta[n] <- intercepttheta[personInd[n]] + betacondtheta[1]*Xcond[n,1] + betacondtheta[2]*Xcond[n,2] + betacondtheta[3]*Xcond[n,3];

}

}

model {

// Regression

interceptnu ~ normal(X*betanu, sigmanu);

intercepttheta ~ normal(X*betatheta, sigmatheta);

// Priors

sigmanu ~ normal(0,10);

sigmatheta ~ normal(0,10);

betanu ~ normal(0,10);

betatheta ~ normal(0,10);

betacondnu ~ normal(0,10);

betacondtheta ~ normal(0,10);

// Likelihood

for (n in 1:N) {

Y[n] ~ normal(nu[n]/theta[n], 1/theta[n]);

}

}
