# Supplementary material for: A Novel Hierarchical LATER Process Model: Evaluating Latent Sources of Variation in Reaction Times of Adult Daily Smokers
Source: Front Psychiatry. 2019 Jul 5;10:474. doi: 10.3389/fpsyt.2019.00474 (PMC6624441; doi:10.3389/fpsyt.2019.00474)
Supplement: Supplementary file 2 [file Table_1.docx]

**Appendix B**

Table S1. Summary of accretion and caution parameters for model covariates. Note. Mean and SD are posterior mean and standard deviation. FTND = Fagerstrom Test for Nicotine Dependence. CrI = credibility interval; SD = Standard Deviation.

|  | Predictor | Mean | SD | 95% Crl |
| --- | --- | --- | --- | --- |
| β_1Accretion_ | Age | -0.0589 | 0.0839 | (-0.2259 , 0.1098) |
| β_2Accretion_ | Age of Smoking Onset | 0.0072 | 0.1222 | (-0.2356 , 0.2517) |
| β_3Accretion_ | FTND | 0.0064 | 0.1361 | (-0.2612 , 0.2837) |
| β_4Accretion_ | Average Cigarettes Per Day | 0.0363 | 0.0834 | (-0.1346 , 0.2033) |
| β_1Caution_ | Age | -0.0798 | -0.0781 | (-0.8302 , 0.6592) |
| β_2Caution_ | Age of Smoking Onset | 0.1369 | 0.1346 | (-0.9271 , 1.2409) |
| β_3Caution_ | FTND | -0.1756 | -0.1746 | (-1.3957 , 1.0342) |
| β_4Caution_ | Average Cigarettes Per Day | 0.1562 | 0.1620 | (-0.6086 , 0.8730) |
